# Tislelizumab for cervical cancer: A retrospective study and analysis of correlative blood biomarkers

**DOI:** 10.3389/fimmu.2023.1113369

**Published:** 2023-02-15

**Authors:** Xiaojing Zheng, Haifeng Gu, Xinping Cao, Baoyue Pan, Huiling Xiang, Mingxiu Ju, Shijie Xu, Min Zheng

**Affiliations:** ^1^ State Key Laboratory of Oncology in South China, Collaborative Innovation Center for Cancer Medicine, Department of Gynecology, Sun Yat-Sen University Cancer Center, Guangzhou, Guangdong, China; ^2^ State Key Laboratory of Oncology in South China, Collaborative Innovation Center for Cancer Medicine, Department of Radiation Oncology, Sun Yat-Sen University Cancer Center, Guangzhou, Guangdong, China

**Keywords:** tislelizumab, cervical cancer, efficacy, adverse events, CRP, CAR, prognosis

## Abstract

**Background:**

Tislelizumab is an anti-programmed cell death 1 (PD-1) monoclonal antibody engineered to minimize binding to Fcγ receptors. It has been used to treat several solid tumors. However, its efficacy and toxicity, and the predictive and prognostic value of baseline hematological parameters in patients with recurrent or metastatic cervical cancer (R/M CC) receiving tislelizumab remain unclear.

**Methods:**

We reviewed 115 patients treated for R/M CC with tislelizumab from March 2020 to June 2022 in our institute. The antitumor activity of tislelizumab was assessed using RECIST v1.1. Associations between the baseline hematological parameters and efficacy of tislelizumab in these patients were analyzed.

**Results:**

With a median follow-up of 11.3 months (range, 2.2–28.7), the overall response rate was 39.1% (95% CI, 30.1–48.2) and the disease control rate was 77.4% (95% CI, 69.6–85.2). The median progression-free survival (PFS) was 19.6 months (95% CI, 10.7 to not reached). The median overall survival (OS) was not reached. Treatment-related adverse events (TRAEs) of any grade occurred in 81.7% of the patients and only 7.0% of the patients experienced grade 3 or 4 TRAEs. Univariate and multivariate regression analyses showed that the level of pretreatment serum C-reactive protein (CRP) was an independent risk factor for the response (complete or partial response) to tislelizumab and the PFS of R/M CC patients treated with tislelizumab (*P* = 0.0001 and *P* = 0.002, respectively). R/M CC patients with elevated baseline CRP levels had a short PFS (*P* = 0.0005). Additionally, the CRP-to-albumin ratio (CAR) was an independent risk factor for the PFS and OS of R/M CC patients treated with tislelizumab (*P* = 0.001 and *P* = 0.031, respectively). R/M CC patients with an elevated baseline CAR had short PFS and OS (*P* < 0.0001 and *P* = 0.0323, respectively).

**Conclusions:**

Tislelizumab showed promising antitumor activity and tolerable toxicity in patients with R/M CC. The baseline serum CRP levels and CAR showed potential for predicting the efficacy of tislelizumab and the prognosis of R/M CC patients receiving tislelizumab.

## Introduction

Cervical cancer (CC) is the fourth most common and lethal female malignancy worldwide. It presents a serious global health challenge, especially in developing countries (1, 2). In China, CC ranks as the second most frequently diagnosed cancer in women (3). Despite advances in vaccination, and screening, approximately 15% of patients are diagnosed with recurrent or metastatic (R/M) disease, with a 5-year survival rate of 15% (4, 5). Platinum-based chemotherapy combined with the anti-angiogenesis agent bevacizumab is the first-line treatment for R/M CC; however, the antitumor response rate is low (2, 6). Therefore, effective therapeutic agents for patients with R/M CC are urgently required.

Immune checkpoint inhibitors (ICIs) have shown promise in the treatment of malignancies. They mainly function by eliminating immunosuppression in the immune microenvironment, as in cases of melanoma, non-small cell lung cancer, and urothelial carcinoma (7). CC is a T-cell inflammatory cancer with high expression levels of programmed cell death 1 (PD-1) and programmed death ligand 1 (PD-L1), especially in advanced or metastatic stages. This indicates that PD-1/PD-L1 inhibitors are potential therapeutic agents (8–10). Recently, several clinical trials have reported exciting outcomes in patients with CC who were administered PD-1/PD-L1 inhibitors. Pembrolizumab has also been approved for the treatment of advanced PD-L1-positive CC (11–13). However, the antitumor response of PD-1/PD-L1 inhibitors have been modest and the economic burden is heavy.

Tislelizumab, a humanized immunoglobulin (Ig) G4 monoclonal antibody with high specificity and affinity for PD-1, was engineered to minimize the binding of Fcγ receptors on macrophages. It reduces antibody-dependent phagocytosis, which is thought to be the mechanism underlying T-cell clearance and resistance to anti-PD-1 therapy (14). Given its favorable antitumor activity and tolerance, tislelizumab has been approved by the U.S. Food and Drug Administration (FDA) for the treatment of esophageal cancer, hepatocellular carcinoma, and gastric/gastroesophageal junction cancer (15). However, the efficacy and safety of tislelizumab in patients with R/M CC has not been adequately evaluated.

Several predictive biomarkers, such as PD-L1 expression, tumor mutational burden (TMB), and microsatellite instability (MSI) status, have been used to select patients who may benefit from ICIs (16–18). However, irrespective of PD-L1 expression levels, promising efficacy has been reported for immunotherapy in CC patients and the proportion of CC patients with high TMB or MSI was also very low (13, 19). Furthermore, tumor tissue samples can be difficult to obtain to measure the above biomarkers. Therefore, it is important to develop clinically and economically feasible predictive biomarkers that can be used to identify patients who may benefit from anti-PD-1 therapy. There is emerging evidence suggesting that several hematological parameters obtained from routine blood tests can reflect inflammation and nutritional status and thereby, play an essential role in predicting immunotherapy efficacy. Some examples of these parameters are C-reactive protein (CRP) concentration, CRP-to-albumin ratio (CAR), and neutrophil-to-lymphocyte ratio (NLR) (20). Elevated CAR and pretreatment CRP levels predict poor antitumor responses and clinical outcomes for the treatment of various malignancies with PD-1 inhibitors (21–23). However, the predictive value of these blood biomarkers in patients with CC receiving PD-1 inhibitors has not been assessed.

Thus, we conducted a retrospective study to assess the efficacy and safety of tislelizumab in patients with R/M CC. In addition, we investigated the predictive and prognostic value of clinical characteristics and hematological parameters in patients who underwent tislelizumab therapy.

## Materials and methods

### Patient selection and procedures

Patients with histologically and radiographically confirmed R/M CC who underwent at least three cycles of tislelizumab (BeiGene, China) treatment from March 2020 to June 2022 at the Sun Yat-sen University Cancer Center were enrolled retrospectively. Some patients received tislelizumab monotherapy, whereas others received tislelizumab combined with a platinum-based chemotherapy, anti-angiogenesis therapy (bevacizumab or apatinib), or local radiotherapy. Tislelizumab was administered at a dose of 200 mg every 3 weeks. The medical history, laboratory results, radiological results, and prior treatments before receiving tislelizumab were reviewed retrospectively for each patient. The antitumor response was evaluated using computed tomography (CT), magnetic resonance imaging, or positron emission tomography/CT at baseline, 5–20 weeks after treatment initiation, and approximately every 3 months thereafter, in accordance with the Response Evaluation Criteria in Solid Tumors (RECIST) v1.1.

### Data collection

The following characteristic clinical data were collected: age, International Federation of Gynecology and Obstetrics (FIGO) stage (version 2018), Eastern Cooperative Oncology Group performance status (ECOG PS), histological type and grade, location of metastases, target lesion size, p16 expression status, dexamethasone use, body mass index (BMI), treatment modality, hematological parameters from routine blood tests (lymphocyte, neutrophil, platelet, and monocyte counts and albumin and CRP concentration), disease progression date, and last follow-up status. All of the follow-up sessions were conducted from the initiation of tislelizumab treatment until September 30, 2022.

The blood biomarker levels were measured within a week prior to the onset of tislelizumab treatment. The CAR, NLR, platelet-to-lymphocyte ratio (PLR), monocyte-to-lymphocyte ratio (MLR), prognostic nutritional index (PNI) value, systemic immune-inflammation index (SII) value, and geriatric nutritional risk index (GNRI) value were calculated based on the absolute counts of serum lymphocytes, neutrophils, platelets, and monocytes plus levels of serum albumin and CRP using the following formulae: CAR = serum CRP concentration/serum albumin concentration; NLR = absolute neutrophil count (10^9^/L)/total lymphocyte count (10^9^/L); PLR = absolute platelet count (10^9^/L)/total lymphocyte count (10^9^/L); MLR = absolute monocyte count (10^9^/L)/total lymphocyte count (10^9^/L); PNI = serum albumin concentration (g/L) + 5 × total lymphocyte count (10^9^/L); SII = absolute platelet count (10^9^/L) × NLR; and GNRI = 1.489 × serum albumin concentration (g/dL) + 41.7 × (current body weight/ideal body weight). The optimal cutoff values for the above hematological parameters were calculated individually using a receiver operating characteristic (ROC) curve for an antitumor objective response.

Following RECIST v1.1, the antitumor response to tislelizumab was classified as a complete response (CR), a partial response (PR), stable disease (SD), or progressive disease (PD). The primary end point was the objective response rate (ORR), defined as the proportion of patients who achieved a CR or PR. We set three secondary end points: the disease control rate (DCR), defined as the proportion of patients who achieved CR, PR, or SD; progression-free survival (PFS), defined as the time from the first cycle of tislelizumab treatment to disease progression or death from any cause; and overall survival (OS), defined as the time from the first cycle of tislelizumab treatment to death from any cause. Adverse events (AEs) were recorded based on the National Cancer Institute Common Terminology Criteria for Adverse Events, v4.0. This study was approved by the Institutional Review Board of Sun Yat-sen University Cancer Center (B2022-715-01).

### Statistical analysis

Statistical analyses were conducted using IBM SPSS v25.0 (IBM, Armonk, NY, U.S.A.) and GraphPad Prism v7.0 (GraphPad Software, CA, U.S.A.). ROC curve analysis was performed to determine the optimal cutoff values for predicting the antitumor response (CR or PR) and the area under curve (AUC) was used to evaluate the predictive value of histological type, CRP levels, and PNI and GNRI values. Pearson’s chi-square test and Fisher’s exact test were performed to determine the relationships between the clinical characteristics and the antitumor response (CR or PR), where appropriate. Logistic regression analysis was used to assess independent predictors of the antitumor response and Cox proportional hazards regression analysis was used to determine the independent risk factors for clinical outcomes. The Kaplan–Meier method was used to plot the survival curves and the log-rank test was used to analyze survival rates. *P* < 0.05 was considered statistically significant. The nomogram for predicting the PFS and OS of R/M CC patients receiving tislelizumab with CRP and CAR was formulated based on the final Cox proportional hazard regression model and conducted using the package of rms in R version 3.5.1.

## Results

### Patient characteristics

We enrolled and treated 115 patients during the study period. The median follow-up was 11.3 months (range, 2.2–28.7) and the median duration of treatment was 4.9 months (range, 1.2–24.5). All the patients were eligible for evaluations of efficacy and safety. The median age was 54.0 years (range, 32–70). The ECOG PS was 0 for 51 cases (44.3%) and not less than 1 for 64 cases (55.7%). Squamous cell carcinoma (SCC) was the most common pathological subtype (75.7%). The median size of the target lesion was 58.5 mm (range, 9–358) and most patients (44.3%) were treated with tislelizumab as the first-line therapy. Thirty patients (26.1%) received tislelizumab combined with radiotherapy, 30 patients (26.1%) received tislelizumab combined with bevacizumab, and 14 patients (12.2%) received tislelizumab combined with apatinib. The median number of cycles of tislelizumab treatment was six (range, 3–34). In addition, BMI and hematological parameters were recorded and the optimal cutoff values of blood biomarkers were determined using the ROC curve for the antitumor response (CR + PR). Detailed baseline characteristics of the patients are presented in **Table 1**.

**Table 1:** Baseline characteristics (n = 115).

Characteristics	No. of patients	%
Age, years, median (range)	54.0 (32-70)	
FIGO stage at initial diagnosis		
I	23	20.0
II	19	16.5
III	53	46.1
IV	15	13.0
Unknown	5	4.3
Time from initial cancer diagnosis to study enrollment, months, median (range)	25.0 (1.3-206.1)	
ECOG PS		
0	51	44.3
≥ 1	64	55.7
Histology		
SCC	87	75.7
Adenocarcinoma	19	16.5
Adenosquamous carcinoma	2	1.7
Other	5	4.3
Unknown	2	1.7
Histological grade		
G1	4	3.5
G2	44	38.3
G3	50	43.5
Unknown	17	14.8
Location of metastases		
Lung	43	37.4
Liver	8	7.0
Pelvis	19	16.5
Lymph node		
Distant lymph nodes	41	35.7
Para-aortic lymph nodes	26	22.6
Pelvic lymph nodes	31	27.0
Bone	19	16.5
Pleura	4	3.5
Bladder	6	5.2
Spleen	1	0.9
Other	24	20.9
Target lesion size, mm, median (range)	58.5 (9-358)	
Previous radiotherapy	83	72.2
Adjuvant radiotherapy	43	37.4
Curative radiotherapy	37	32.2
Palliative radiotherapy	3	2.6
No. of previous systemic therapies		
0	51	44.3
1	48	41.7
≥ 2	16	13.9
Previous platinum	99	86.1
Previous paclitaxel	86	74.8
Previous bevacizumab	27	23.5
Previous apatinib	17	14.8
Previous ICIs	17	14.8
P16 expression		
Positive	53	46.1
Negative	10	8.7
Unknown	52	45.2
Tislelizumab monotherapy	2	1.7
Combined with radiotherapy	30	26.1
Combined with bevacizumab	30	26.1
Combined with apatinib	14	12.2
Dexamethasone use	46	40.0
Cycles of tislelizumab, median (range)	6 (3-34)	
BMI		
≤ 24.9	66	57.4
> 24.9	39	33.9
Lymphocyte, × 10^9^/L, median (range)	1.01 (0.21-3.09)	
Neutrophil, × 10^9^/L, median (range)	3.98 (1.26-12.62)	
Monocyte, × 10^9^/L, median (range)	0.35 (0.14-1.12)	
Platelet, × 10^9^/L, median (range)	249 (79-574)	
CRP, mg/L, median (range)	5.87 (0.21-215.39)	
Albumin, g/L, median (range)	43.0 (26.1-50.4)	
CAR		
≤ 0.085	46	40.0
> 0.085	69	60.0
NLR		
≤ 4.84	73	63.5
> 4.84	42	36.5
PLR		
≤ 169.57	30	26.1
> 169.57	85	73.9
MLR		
≤ 0.27	39	33.9
> 0.27	76	66.1
PNI		
≤ 49.05	63	54.8
> 49.05	52	45.2
SII		
≤ 980.0	66	57.4
> 980.0	49	42.6
GNRI		
≤ 117.1	89	77.4
> 117.1	16	13.9

Variables are expressed as number of patients (%).

No., number; FIGO stage, Federation of Gynecology and Obstetrics stage; ECOG PS, Eastern Cooperative Oncology Group performance status; SSC, squamous cell carcinoma, ICIs, immune checkpoint inhibitors; BMI, body mass index; CRP, C-reactive protein; CAR, CRP-to-albumin ratio; NLR, neutrophil-to-lymphocyte ratio; PLR, platelet-to-lymphocyte ratio; MLR, monocyte-to-lymphocyte ratio; PNI, prognostic nutritional index; SII, systemic immune-inflammation index; GNRI, geriatric nutritional risk index.

### Antitumor activity

**Figure 1:**
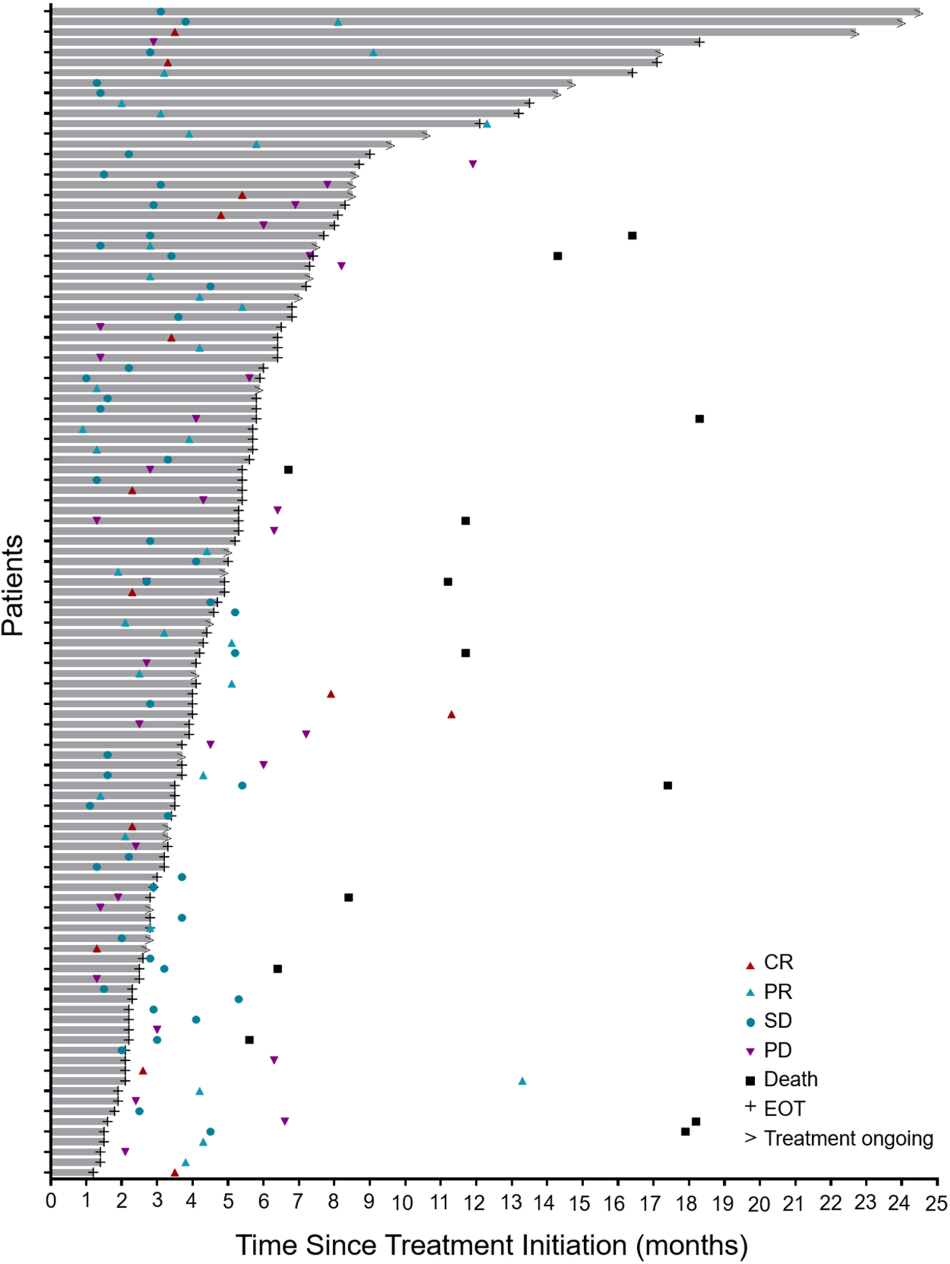
Antitumor activity. Duration of the responses of all treated patients (n = 115). The length of each bar represents the duration of Tislelizumab treatment for each patient. CR, complete response; PR, partial response; SD, stable disease; PD, progressive disease; EOT, end of treatment.

**Figure 2:**
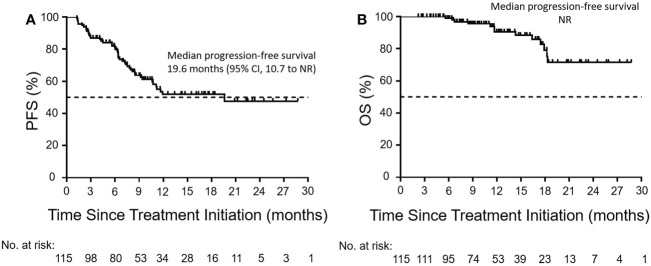
Kaplan–Meier curves of PFS and OS. Kaplan–Meier curves of **(A)** PFS and **(B)** OS in the enrolled population (n = 115). The Kaplan–Meier analysis was used to plot the survival curves. The dashed line indicates a PFS or OS rate of 50%. PFS, progression-free survival; OS, overall survival; CI, confidence interval; NR, not reached.

During the treatment period, the best overall response of each patient was recorded. The median time to response was 3.1 months (range, 0.9–13.3; **Figure 1**). As shown in **Table 2**, 13 patients (11.3%) achieved a CR, 32 patients (27.8%) attained a PR, 44 (38.3%) experienced SD, and 26 (22.6%) developed PD. The ORR was 39.1% (95% confidence interval [CI], 30.1–48.2) and the DCR was 77.4 (95% CI, 69.6–85.2). At the data cutoff point, 46 patients (40%) either developed PD or died. The median PFS was 19.6 months (95% CI, 10.7 to not reached; **Figure 2A**). OS events occurred in 13 patients (11.3%). The median OS was not reached (**Figure 2B**).

**Table 2:** Antitumor activity assessed by RECIST Version 1.1.

Antitumor Activity	n = 115
Best overall response, No. (%)	
CR	13 (11.3)
PR	32 (27.8)
SD	44 (38.3)
PD	26 (22.6)
ORR, No. (%)	45 (39.1)
95% CI	30.1-48.2
DCR, No. (%)	89 (77.4)
95% CI	69.6-85.2

Variables are expressed as number of patients (%).

No., number; CR, complete response; PR, partial response; SD, stable disease; PD, progressive disease; ORR, objective response rate; DCR, disease control rate; CI, confidence interval; RECIST, the Response Evaluation Criteria in Solid Tumors.

### Safety

Ninety-four patients (81.7%) experienced at least one treatment-related AE (TRAE), the most common of which were anemia (47.0%), hypothyroidism (15.7%), and thrombocytopenia (12.2%; **Table 3**). Most of the observed AEs were grade 1–2, and grade 4 TRAEs and treatment-related deaths were not observed. Eight patients (7.0%) experienced grade 3 TRAEs, namely thrombocytopenia (4 patients, 3.5%), hypertension (2 patients, 1.7%), and neutropenia (2 patients, 1.7%). Twelve patients (10.4%) discontinued treatment because of the TRAEs. Overall, the treatment of patients with R/M CC using tislelizumab appeared to be safe.

**Table 3:** Treatment-related adverse events in all treated patients.

	No. (%) of patients (n = 115)			
Adverse Event	Total	Grade 1	Grade 2	Grade 3
Anemia	54 (47.0)	32 (27.8)	22 (19.1)	0 (0)
Hypothyroidism	18 (15.7)	8 (7.0)	10 (8.7)	0 (0)
Thrombocytopenia	14 (12.2)	3 (2.6)	7 (6.1)	4 (3.5)
Hand-foot syndrome	13 (11.3)	8 (7.0)	5 (4.3)	0 (0)
Anorexia	12 (10.4)	5 (4.3)	7 (6.1)	0 (0)
Hypertension	10 (8.7)	3 (2.6)	5 (4.3)	2 (1.7)
Diarrhea	8 (7.0)	4 (3.5)	2 (1.7)	0 (0)
Fatigue	8 (7.0)	8 (7.0)	0 (0)	0 (0)
Neutropenia	8 (7.0)	2 (1.7)	4 (3.5)	2 (1.7)
Nausea	7 (6.1)	2 (1.7)	5 (4.3)	0 (0)
Vomiting	7 (6.1)	1 (0.9)	6 (5.2)	0 (0)
ALT elevation	6 (5.2)	6 (5.2)	0 (0)	0 (0)
Rash	5 (4.3)	2 (1.7)	3 (2.6)	0 (0)
Headache/dizziness	5 (4.3)	5 (4.3)	0 (0)	0 (0)
Hoarseness	4 (3.5)	4 (3.5)	0 (0)	0 (0)
AST elevation	4 (3.5)	4 (3.5)	0 (0)	0 (0)
Limb numbness	4 (3.5)	3 (2.6)	1 (0.9)	0 (0)
Constipation	3 (2.6)	2 (1.7)	1 (0.9)	0 (0)
Palpitation/chest pain	3 (2.6)	2 (1.7)	1 (0.9)	0 (0)
Dental ulcer	2 (1.7)	0 (0)	2 (1.7)	0 (0)
Immune hepatitis	2 (1.7)	0 (0)	2 (1.7)	0 (0)
Dry mouth	2 (1.7)	2 (1.7)	0 (0)	0 (0)
Lumbago	2 (1.7)	1 (0.9)	1 (0.9)	0 (0)
Creatine phosphokinase elevation	2 (1.7)	1 (0.9)	1 (0.9)	0 (0)
lactate dehydrogenase elevation	1 (0.9)	1 (0.9)	0 (0)	0 (0)
γ-glutamyltransferase elevation	1 (0.9)	1 (0.9)	0 (0)	0 (0)
Hypercholesterolemia	1 (0.9)	1 (0.9)	0 (0)	0 (0)
Hypoalbuminemia	1 (0.9)	1 (0.9)	0 (0)	0 (0)
Proteinuria	1 (0.9)	1 (0.9)	0 (0)	0 (0)
Fever	1 (0.9)	0 (0)	1 (0.9)	0 (0)
Fistula	1 (0.9)	0 (0)	1 (0.9)	0 (0)
Gingival hemorrhage	1 (0.9)	1 (0.9)	0 (0)	0 (0)
Back pain	1 (0.9)	1 (0.9)	0 (0)	0 (0)
Hyperthyroidism	1 (0.9)	0 (0)	1 (0.9)	0 (0)

Variables are expressed as number of patients (%).

No., number; ALT, alanine transaminase; AST, aspartate transaminase.

### Relationship between antitumor response and clinical characteristics

**Figure 3:**
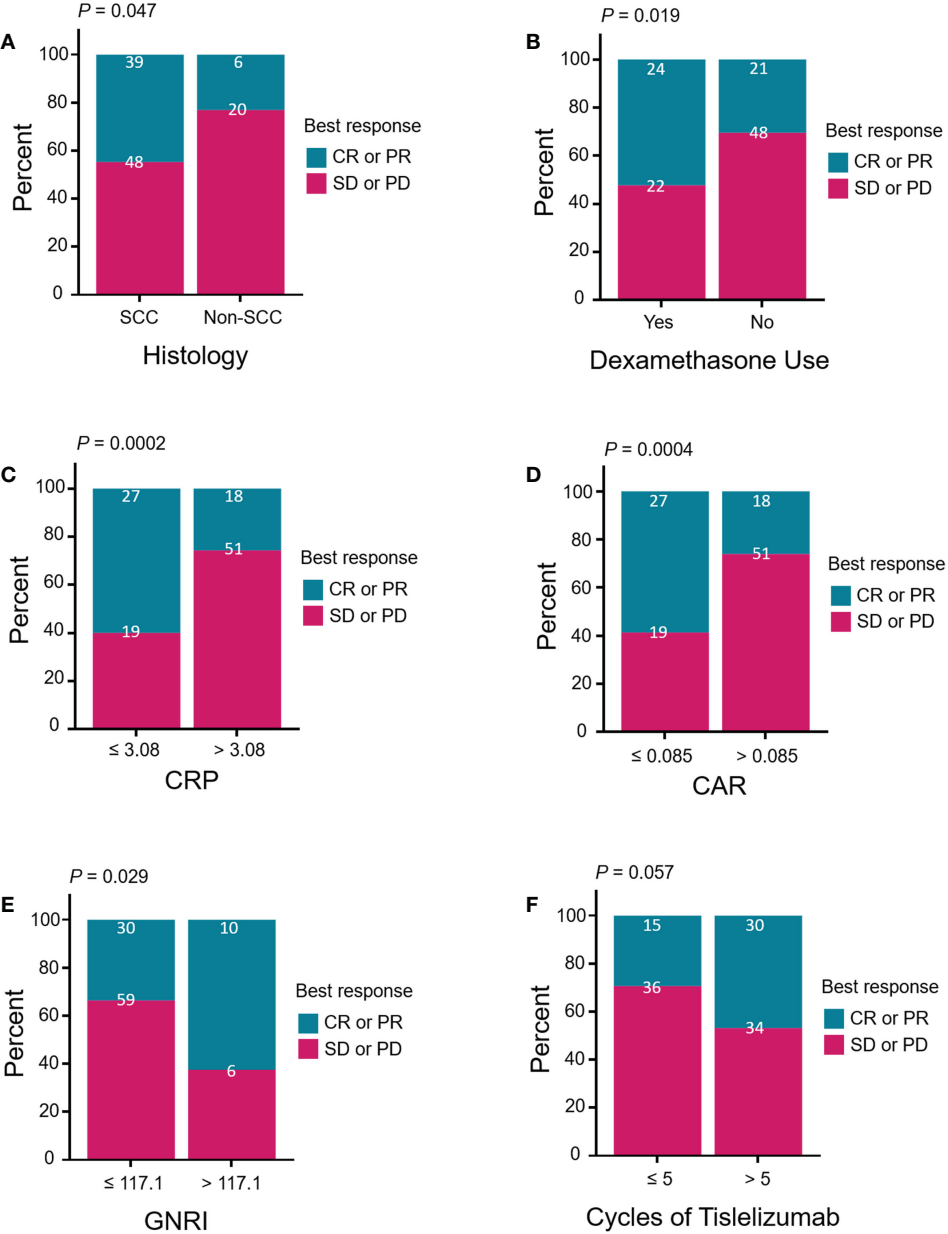
Treatment response of patients stratified by **(A)** histology (squamous vs. non-squamous), **(B)** dexamethasone use (yes vs. no), **(C)** CRP (≤ 3.08 vs. > 3.08, mg/L), **(D)** CAR (≤ 0.085 vs. > 0.085), **(E)** GNRI (≤ 117.1 vs. > 117.1), and **(F)** cycles of tislelizumab (≤ 5 vs. > 5). Treatment response was divided by CR or PR (blue group) versus SD or PD (red group). Pearson’s chi-square test and Fisher’s exact test were performed to determine the relationships between the above clinical characteristics and treatment response. CR, complete response; PR, partial response; SD, stable disease; PD, progressive disease; SSC, squamous cell carcinoma; CRP, C-reactive protein; CAR, CRP-to-albumin ratio; GNRI, geriatric nutritional risk index.

The associations between the antitumor response (CR + PR) and clinical characteristics are summarized in **Table 4**. The ORR was 44.8% (39/87) in SCC and 23.1% (6/26) in non-SCC patients (*P* = 0.047, **Figure 3A**). The ORR for using dexamethasone at the time of study initiation, which mainly to alleviate chemotherapy-induced nausea and vomiting, was higher than the ORR for not using dexamethasone (52.2% vs. 30.0%, *P* = 0.019; **Figure 3B**). The high levels of baseline serum CRP and CAR were significantly associated with low ORRs (CRP ≤ 3.08 vs. CRP > 3.08, 58.7% vs. 26.1%, *P* = 0.0002; CAR ≤ 0.085 vs. CAR > 0.085, 58.7% vs. 26.1%, *P* = 0.0004; **Figures 3C, D**). A high GNRI value was significantly associated with a high ORR (GNRI ≤ 117.1 vs. GNRI > 117.1, 33.7% vs. 62.5%, *P* = 0.029; **Figure 3E**). Patients treated with more than five cycles of tislelizumab tended to have a high ORR (46.9% vs. 29.4%, *P* = 0.057; **Figure 3F**). Moreover, previous treatment with platinum, paclitaxel, or apatinib before enrollment may have affected the antitumor response of tislelizumab.

**Table 4:** Correlation between the antitumor response and clinicopathological factors in patients treated with tislelizumab.

Characteristics	Antitumor Activity		*P* value
	CR+PR	SD+PD	
Age, years			
≤ 55	29 (64.4)	40 (57.1)	0.425
> 55	16 (35.6)	30 (42.9)	
ECOG PS			
0	24 (53.3)	27 (38.6)	0.120
≥ 1	21 (46.7)	43 (61.4)	
FIGO stage at initial diagnosis			
I + II	16 (39.0)	26 (37.7)	0.888
III + IV	25 (61.0)	43 (62.3)	
Time from initial cancer diagnosis to study enrollment, months			
≤ 15.7	9 (23.1)	24 (38.1)	0.115
> 15.7	30 (76.9)	39 (61.9)	
Histology			
SCC	39 (86.7)	48 (70.6)	**0.047**
Non-SCC	6 (13.3)	20 (29.4)	
Histological grade			
G1 + G2	17 (44.7)	31 (51.7)	0.504
G3	21 (55.3)	29 (48.3)	
Target lesion size, mm			
≤ 51	22 (50.0)	22 (33.3)	0.080
> 51	22 (55.0)	44 (66.7)	
Previous platinum			
Yes	34 (75.6)	65 (92.9)	**0.009**
No	11 (24.4)	5 (7.1)	
Previous paclitaxel			
Yes	29 (64.4)	57 (81.4)	**0.041**
No	16 (35.6)	13 (18.6)	
Previous bevacizumab			
Yes	7 (15.6)	20 (28.6)	0.108
No	38 (84.4)	50 (71.4)	
Previous apatinib			
Yes	2 (4.4)	15 (21.4)	**0.012**
No	43 (95.6)	55 (78.6)	
Previous ICIs			
Yes	10 (22.2)	7 (10.0)	0.072
No	35 (77.8)	63 (90.0)	
Previous radiotherapy			
Yes	32 (71.1)	51 (72.9)	0.838
No	13 (28.9)	19 (27.1)	
P16 expression			
Positive	20 (90.9)	33 (80.5)	0.472
Negative	2 (9.1)	8 (19.5)	
Combined with bevacizumab			
Yes	12 (26.7)	18 (25.7)	0.910
No	33 (73.3)	52 (74.3)	
Combined with apatinib			
Yes	3 (6.7)	11 (15.7)	0.148
No	42 (93.3)	59 (84.3)	
Combined with radiotherapy			
Yes	16 (35.6)	14 (20.0)	0.064
No	29 (64.4)	56 (80.0)	
Dexamethasone use			
Yes	24 (53.3)	22 (31.4)	**0.019**
No	21 (46.7)	48 (68.6)	
Cycles of tislelizumab			
≤ 5	15 (33.3)	36 (51.4)	**0.057**
> 5	30 (66.7)	34 (48.6)	
BMI			
≤ 24.9	25 (62.5)	41 (63.1)	0.953
> 24.9	15 (37.5)	24 (36.9)	
Lymphocyte, × 10^9^/L			
≤ 1.33	34 (75.6)	43 (61.4)	0.116
> 1.33	11 (24.4)	27 (38.6)	
Neutrophil, × 10^9^/L			
≤ 7.21	38 (84.4)	65 (92.9)	0.212
> 7.21	7 (15.6)	5 (7.1)	
Monocyte, × 10^9^/L			
≤ 0.35	25 (55.6)	33 (47.1)	0.379
> 0.35	20 (44.4)	37 (52.9)	
Platelet, × 10^9^/L			
≤ 265	31 (68.9)	41 (58.6)	0.264
> 265	14 (31.1)	29 (41.4)	
CRP, mg/L			
≤ 3.08	27 (60.0)	18 (25.7)	**0.0002**
> 3.08	18 (40.0)	52 (74.3)	
CAR			
≤ 0.085	27 (60.0)	19 (27.1)	**0.0004**
> 0.085	18 (40.0)	51 (72.9)	
NLR			
≤ 4.84	25 (55.6)	48 (68.6)	0.157
> 4.84	20 (44.4)	22 (31.4)	
PLR			
≤ 169.57	8 (17.8)	22 (31.4)	0.104
> 169.57	37 (82.2)	48 (68.6)	
MLR			
≤ 0.27	12 (26.7)	27 (38.6)	0.188
> 0.27	33 (73.3)	43 (61.4)	
PNI			
≤ 49.05	29 (64.4)	34 (48.6)	0.095
> 49.05	16 (35.6)	36 (51.4)	
SII			
≤ 980.0	22 (48.9)	44 (62.9)	0.139
> 980.0	23 (51.1)	26 (37.1)	
GNRI			
≤ 117.1	30 (75.0)	59 (90.8)	**0.029**
> 117.1	10 (25.0)	6 (9.2)	

CR, complete response; PR, partial response; SD, stable disease; PD, progressive disease; FIGO stage, Federation of Gynecology and Obstetrics stage; ECOG PS, Eastern Cooperative Oncology Group performance status; SSC, squamous cell carcinoma; ICIs, immune checkpoint inhibitors; BMI, body mass index; CRP, C-reactive protein; CAR, CRP-to-albumin ratio; NLR, neutrophil-to-lymphocyte ratio; PLR, platelet-to-lymphocyte ratio; MLR, monocyte-to-lymphocyte ratio; PNI, prognostic nutritional index; SII, systemic immune-inflammation index; GNRI, geriatric nutritional risk index. Bold values indicate statistical significance.

### Univariate and multivariate analysis of the antitumor response

**Figure 4:**
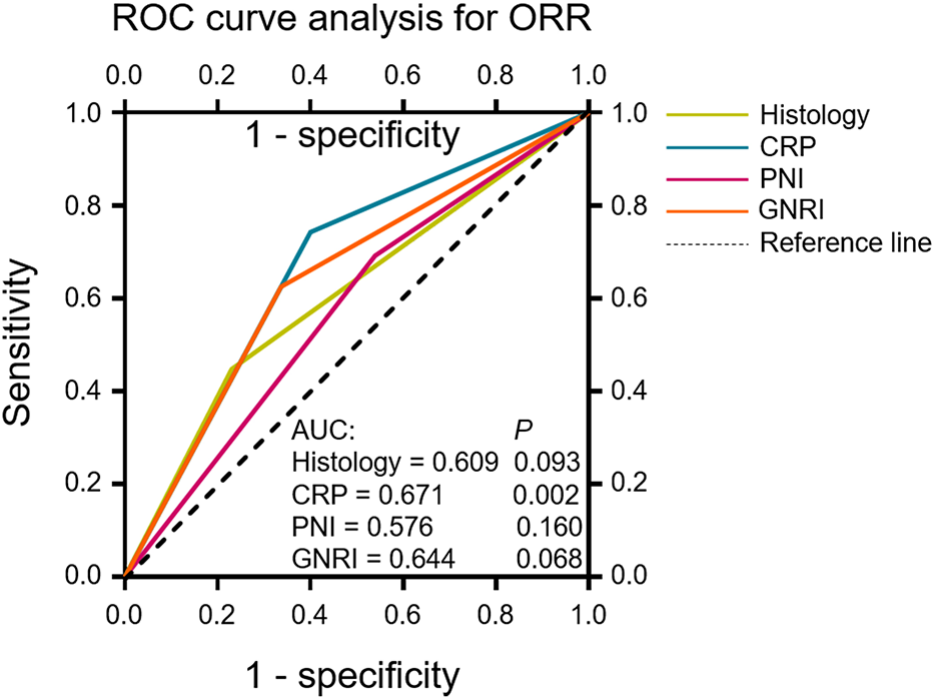
ROC curves evaluating the accuracy of the histological type, CRP, PNI and GNRI for response (CR or PR) prediction in patients who underwent tislelizumab treatment. ROC curve analysis was performed to determine the optimal cutoff values and the AUC was used to evaluate the predictive value of the above clinical characteristics. The reference line indicates an AUC of 0.5. ROC, receiver operating characteristic; ORR, objective response rate; CR, complete response; PR, partial response; CRP, C-reactive protein; PNI, prognostic nutritional index; GNRI, geriatric nutritional risk index; AUC, area under the curve.

The patients’ age, ECOG PS, FIGO stage, histology, target lesion size, drug used in combination with tislelizumab, dexamethasone use, number of tislelizumab treatment cycles, and blood biomarkers were included in univariate logistic regression analyses of the antitumor response (CR + PR; **Table 5**). Dexamethasone use, CRP levels, CAR, and GNRI were significant predictors of the antitumor response to tislelizumab in R/M CC patients. Multivariate logistic regression analysis revealed that the histological type (OR: 4.292, 95% CI: 1.148–16.049, *P* = 0.030), CRP levels (OR: 11.101, 95% CI: 3.233–38.121, *P* = 0.0001), PNI (OR: 7.224, 95% CI: 1.955–26.700, *P* = 0.003), and GNRI (OR: 0.075, 95% CI: 0.014–0.392, *P* = 0.002) were independent predictors of the antitumor response to tislelizumab in R/M CC patients. To determine the most reliable predictor of the antitumor response, an ROC curve was constructed to analyze the relationship between the abovementioned independent predictors and the response to tislelizumab. CRP had a larger AUC than histological type, PNI, and GNRI (AUC = 0.671, *P* = 0.002; **Figure 4**), indicating that CRP is a potential blood biomarker that negatively predicts the antitumor response to tislelizumab in patients with R/M CC.

**Table 5:** Univariate and multivariate analyses of the antitumor response (CR + PR).

	Univariate			Multivariate		
	*P*	OR	95% CI	*P*	OR	95% CI
Age, years (≤ 55 vs. > 55)	0.436	1.359	0.628-2.943			
ECOG PS (0 vs. ≥ 1)	0.121	1.820	0.853-3.884			
FIGO stage (I + II vs. III + IV)	0.888	1.058	0.478-2.342			
Histology (SCC vs. Non-SCC)	0.052	2.708	0.991-7.402	0.030	4.292	1.148-16.049
Histological grade (G1 + G2 vs. G3)	0.504	0.757	0.335-1.712			
Target lesion size, mm (≤ 51 vs. >51)	0.082	2.000	0.915-4.371	0.099		
Combined with bevacizumab (No vs. Yes)	0.910	0.952	0.407-2.229			
Combined with apatinib (No vs. Yes)	0.159	2.610	0.686-9.934			
Combined with radiotherapy (No vs. Yes)	0.067	0.453	0.194-1.056	0.073		
Dexamethasone use (No vs. Yes)	0.021	0.401	0.185-0.869	0.106		
Cycles of tislelizumab (≤ 5 vs. > 5)	0.058	0.472	0.217-1.027	0.805		
Lymphocyte, × 10^9^/L (≤ 1.33 vs. >1.33)	0.119	1.941	0.844-4.464			
Neutrophil, × 10^9^/L (≤ 7.21 vs. > 7.21)	0.159	0.418	0.124-1.408			
Monocyte, × 10^9^/L (≤ 0.35 vs. > 0.35)	0.379	1.402	0.661-2.974			
Platelet, × 10^9^/L (≤ 265 vs. > 265)	0.266	1.566	0.711-3.452			
CRP, mg/L (≤ 3.08 vs. > 3.08)	0.0003	4.333	1.943-9.662	0.0001	11.101	3.233-38.121
CAR (≤ 0.085 vs. > 0.085)	0.001	4.026	1.817-8.923	0.945		
NLR (≤ 4.84 vs. > 4.84)	0.159	0.573	0.264-1.243			
PLR (≤ 169.57 vs. > 169.57)	0.108	0.472	0.189-1.179			
MLR (≤ 0.27 vs. > 0.27)	0.190	0.579	0.256-1.311			
PNI (≤ 49.05 vs. > 49.05)	0.097	1.919	0.889-4.143	0.003	7.224	1.955-26.700
SII (≤ 980.0 vs. > 980.0)	0.141	0.565	0.264-1.208			
GNRI (≤ 117.1 vs. > 117.1)	0.035	0.305	0.101-0.920	0.002	0.075	0.014-0.392

CR, complete response; PR, partial response; OR, odds ratio; CI, confidence interval; FIGO stage, Federation of Gynecology and Obstetrics stage; ECOG PS, Eastern Cooperative Oncology Group performance status; SSC, squamous cell carcinoma; CRP, C-reactive protein; CAR, CRP-to-albumin ratio; NLR, neutrophil-to-lymphocyte ratio; PLR, platelet-to-lymphocyte ratio; MLR, monocyte-to-lymphocyte ratio; PNI, prognostic nutritional index; SII, systemic immune-inflammation index; GNRI, geriatric nutritional risk index.

### Prognostic analysis of clinical factors

To determine the potential factors predictive of the long-term efficacy of tislelizumab in R/M CC patients, univariate and multivariate analyses were performed for PFS and OS. Univariate Cox proportional analysis revealed that high levels of baseline serum CRP (hazard ratio [HR]: 3.373, 95% CI: 1.623–7.007, *P* = 0.001) and CAR (HR: 4.078, 95% CI: 1.898–8.760, *P* = 0.0003) were significantly negative prognostic factors of PFS for patients with R/M CC who were administered tislelizumab (**Table 6**). CAR (HR: 4.510, 95% CI: 0.994–20.455, *P* = 0.051) and PLR (HR: 0.336, 95% CI: 0.112–1.004, *P* = 0.051) were predictors of OS (**Table 7**). Multivariate analysis demonstrated that CRP (HR: 3.200, 95% CI: 1.534–6.676, *P* = 0.002) and CAR (HR: 3.831, 95% CI: 1.776–8.263, *P* = 0.001) were independent predictors of PFS (**Table 6**). A high CAR (HR: 5.388, 95% CI: 1.163–24.972, *P* = 0.031) and low PLR (HR: 0.273, 95% CI: 0.089–0.840, *P* = 0.024) were independent negative prognostic factors for the OS of patients with R/M CC who were administered tislelizumab (**Table 7**).

**Table 6:** Univariate and multivariate analyses of PFS.

	Univariate			Multivariate		
	*P*	HR	95% CI	*P*	HR	95% CI
Age, years (≤ 55 vs. > 55)	0.496	0.810	0.441-1.486			
ECOG PS (0 vs. ≥ 1)	0.442	1.262	0.698-2.281			
FIGO stage (I + II vs. III + IV)	0.320	1.381	0.731-2.608			
Histology (SCC vs. Non-SCC)	0.491	1.263	0.650-2.456			
Histological grade (G1 + G2 vs. G3)	0.423	0.767	0.402-1.466			
Target lesion size, mm (≤ 51 vs. >51)	0.210	1.479	0.802-2.727			
Combined with bevacizumab (No vs. Yes)	0.307	1.388	0.739-2.608			
Combined with apatinib (No vs. Yes)	0.465	0.706	0.277-1.797			
Combined with radiotherapy (No vs. Yes)	0.590	0.837	0.439-1.597			
Dexamethasone use (No vs. Yes)	0.708	0.893	0.494-1.615			
Cycles of tislelizumab (≤ 5 vs. > 5)	0.774	0.918	0.512-1.646			
Lymphocyte, × 10^9^/L (≤ 1.33 vs. >1.33)	0.685	1.136	0.613-2.107			
Neutrophil, × 10^9^/L (≤ 7.21 vs. > 7.21)	0.553	1.326	0.523-3.363			
Monocyte, × 10^9^/L (≤ 0.35 vs. > 0.35)	0.070	1.719	0.957-3.089	0.366		
Platelet, × 10^9^/L (≤ 265 vs. > 265)	0.903	1.038	0.570-1.891			
CRP, mg/L (≤ 3.08 vs. > 3.08)	0.001	3.373	1.623-7.007	0.002	3.200	1.534-6.676
CAR (≤ 0.085 vs. > 0.085)	0.0003	4.078	1.898-8.760	0.001	3.831	1.776-8.263
NLR (≤ 4.84 vs. > 4.84)	0.821	0.933	0.512-1.702			
PLR (≤ 169.57 vs. > 169.57)	0.185	0.659	0.355-1.220			
MLR (≤ 0.27 vs. > 0.27)	0.339	1.359	0.724-2.549			
PNI (≤ 49.05 vs. > 49.05)	0.772	0.917	0.509-1.650			
SII (≤ 980.0 vs. > 980.0)	0.949	0.981	0.547-1.760			
GNRI (≤ 117.1 vs. > 117.1)	0.062	0.259	0.063-1.070	0.106		

PFS, progression-free survival; HR, hazard ratio; CI, confidence interval; FIGO stage, Federation of Gynecology and Obstetrics stage; ECOG PS, Eastern Cooperative Oncology Group performance status; SSC, squamous cell carcinoma; CRP, C-reactive protein; CAR, CRP-to-albumin ratio; NLR, neutrophil-to-lymphocyte ratio; PLR, platelet-to-lymphocyte ratio; MLR, monocyte-to-lymphocyte ratio; PNI, prognostic nutritional index; SII, systemic immune-inflammation index; GNRI, geriatric nutritional risk index.

**Table 7:** Univariate and multivariate analyses of OS.

	Univariate			Multivariate		
	*P*	HR	95% CI	*P*	HR	95% CI
Age, years (≤ 55 vs. > 55)	0.784	1.165	0.389-3.491			
ECOG PS (0 vs. ≥ 1)	0.672	1.273	0.416-3.893			
FIGO stage (I + II vs. III + IV)	0.628	0.763	0.256-2.275			
Histology (SCC vs. Non-SCC)	0.837	0.873	0.240-3.176			
Histological grade (G1 + G2 vs. G3)	0.560	1.385	0.463-4.142			
Target lesion size, mm (≤ 51 vs. >51)	0.362	1.732	0.532-5.637			
Combined with bevacizumab (No vs. Yes)	0.718	1.242	0.382-4.038			
Combined with apatinib (No vs. Yes)	0.740	1.248	0.337-4.615			
Combined with radiotherapy (No vs. Yes)	0.132	0.313	0.069-1.416			
Dexamethasone use (No vs. Yes)	0.515	0.675	0.207-2.200			
Cycles of tislelizumab (≤ 5 vs. > 5)	0.229	0.511	0.171-1.524			
Lymphocyte, × 10^9^/L (≤ 1.33 vs. >1.33)	0.851	0.884	0.243-3.220			
Neutrophil, × 10^9^/L (≤ 7.21 vs. > 7.21)	0.900	0.878	0.114-6.777			
Monocyte, × 10^9^/L (≤ 0.35 vs. > 0.35)	0.487	1.476	0.492-4.431			
Platelet, × 10^9^/L (≤ 265 vs. > 265)	0.654	1.285	0.429-3.850			
CRP, mg/L (≤ 3.08 vs. > 3.08)	0.129	2.732	0.747-9.986			
CAR (≤ 0.085 vs. > 0.085)	0.051	4.510	0.994-20.455	0.031	5.388	1.163-24.972
NLR (≤ 4.84 vs. > 4.84)	0.980	0.986	0.322-3.020			
PLR (≤ 169.57 vs. > 169.57)	0.051	0.336	0.112-1.004	0.024	0.273	0.089-0.840
MLR (≤ 0.27 vs. > 0.27)	0.993	0.995	0.324-3.052			
PNI (≤ 49.05 vs. > 49.05)	0.579	1.363	0.457-4.059			
SII (≤ 980.0 vs. > 980.0)	0.911	0.940	0.315-2.800			
GNRI (≤ 117.1 vs. > 117.1)	0.462	0.042	0.000-194.806			

OS, overall survival; HR, hazard ratio; CI, confidence interval; FIGO stage, Federation of Gynecology and Obstetrics stage; ECOG PS, Eastern Cooperative Oncology Group performance status; SSC, squamous cell carcinoma; CRP, C-reactive protein; CAR, CRP-to-albumin ratio; NLR, neutrophil-to-lymphocyte ratio; PLR, platelet-to-lymphocyte ratio; MLR, monocyte-to-lymphocyte ratio; PNI, prognostic nutritional index; SII, systemic immune-inflammation index; GNRI, geriatric nutritional risk index.

**Figure 5:**
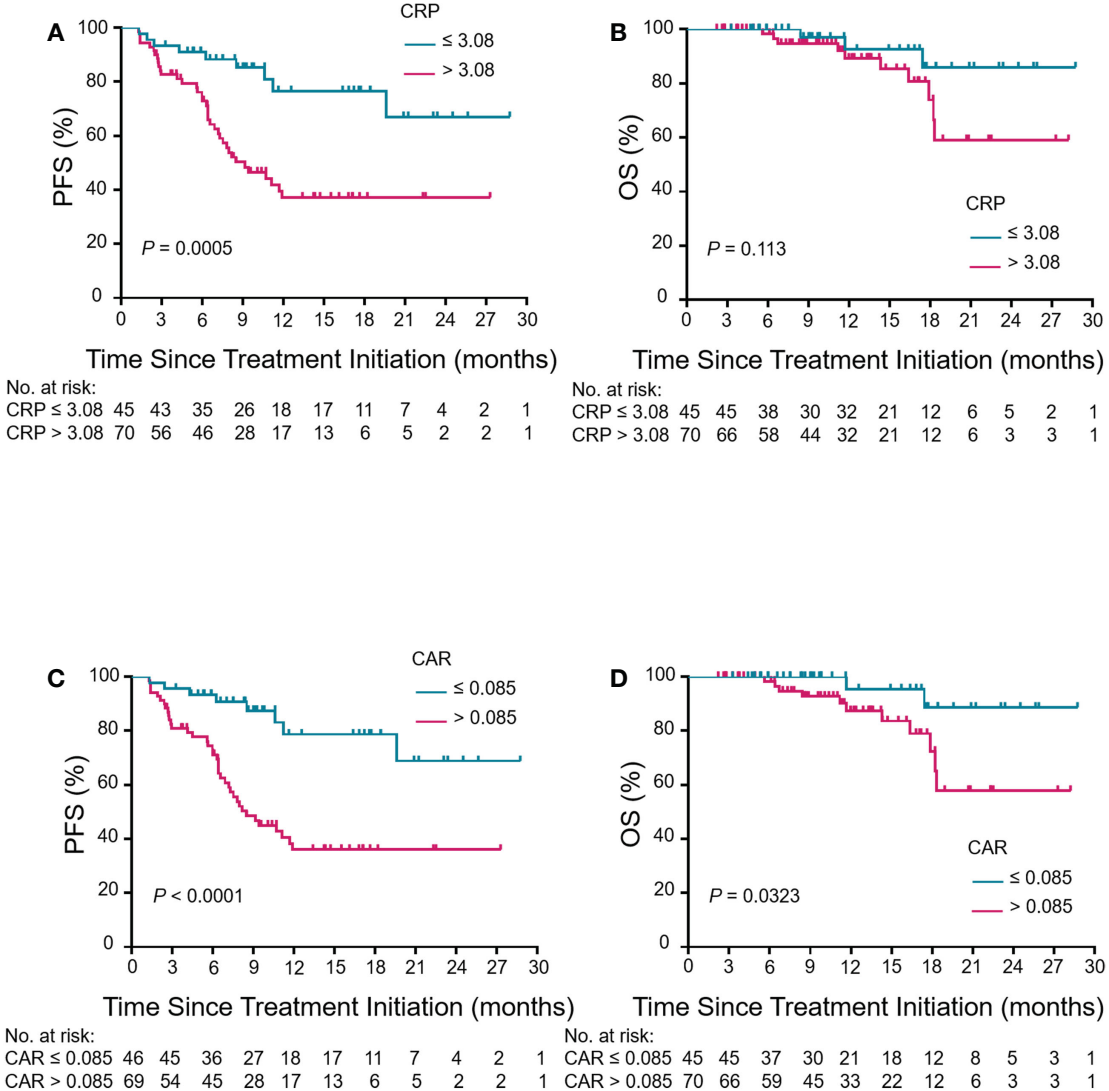
Association between pretreatment CRP and CAR levels and clinical outcomes in patients with R/M CC who underwent tislelizumab treatment. Kaplan–Meier curves of PFS and OS for CRP **(A, B)** and CAR **(C, D)** in the enrolled population. Kaplan–Meier analysis and log-rank tests were used for comparison between low-level group (blue group) and high-level group (red group) of CRP and CAR in PFS and OS. CRP, C-reactive protein; CAR, CRP-to-albumin ratio; PFS, progression-free survival; OS, overall survival; R/M CC, recurrent or metastatic cervical cancer.

**Figure 6:**
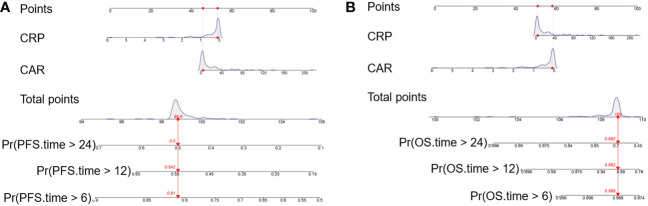
Nomogram model for predicting the **(A)** PFS and **(B)** OS of R/M CC patients receiving tislelizumab with CRP and CAR. The nomogram was formulated based on the final Cox proportional hazard regression model and conducted using the package of rms in R. And it was used summing the points identified on the points scale for each variable. Red lines and dots are drawn upward to determine the points received by each variable. The total points projected on the bottom scales indicate the probability of 6-month, 12-month and 24-month survival (PFS and OS). PFS, progression-free survival; OS, overall survival; R/M CC, recurrent or metastatic cervical cancer; CRP, C-reactive protein; CAR, CRP-to-albumin ratio; Pr, probability.

Kaplan-Meier curves based on baseline CRP, CAR, GNRI and PLR levels were shown in **Figure 5** and **Figure S1**. An elevated CRP level was significantly correlated with poor PFS (*P* = 0.0005, **Figure 5A**). Patients with a high pretreatment CAR had long PFS and OS (*P* < 0.0001 and *P* = 0.0323, **Figures 5C, D**). The PFS rate was significantly higher for the high-GNRI group than the low-GNRI group (*P* = 0.0442, **Figure S1A**). The OS rate was also significantly higher for the high-PLR group than the low-PLR group (*P* = 0.0401, **Figure S1D**). On the basis of the significant variables, CRP and CAR, a fitting model was presented with a nomogram to predict the prognosis of R/M CC patients receiving tislelizumab (**Figures 6A, B**). Each level of the variables was assigned with a specific point on the scale. By summing the points from each variable, the total point was obtained for the individual patients. 6-month, 12-month and 24-month PFS and OS probability can be predicted by projecting the total points to the total score scale of the nomogram. Overall, we found that high levels of pretreatment CRP and CAR predicted poor survival outcomes in patients with R/M CC who were administered tislelizumab.

## Discussion

Recently, ICIs have shown great potential for treating R/M CC. Based on the encouraging antitumor activity and tolerable TRAEs, pembrolizumab was the first immunotherapeutic agent approved by the U.S. FDA for the treatment of patients with R/M CC and high expression levels of PD-L1 following chemotherapy (5). In June 2022, following promising results of a clinical trial (NCT04380805), cadonilimab, a PD-1/cytotoxic T lymphocyte antigen 4 bi-specific antibody, was approved in China to treat patients with R/M CC who had progressed or following platinum-based chemotherapy. However, the prices of the two drugs mentioned above are extremely high and most patients cannot afford them. Thus, more cost-efficient ICIs should be urgently developed.

Tislelizumab, a PD-1 inhibitor, shows high affinity for PD-1, with a 100-fold slower off-rate than pembrolizumab (24). Several clinical trials have reported encouraging antitumor activity and manageable TRAEs in patients with advanced solid tumors who underwent tislelizumab treatment (25, 26). Furthermore, tislelizumab has been included in the medical insurance catalogue in China, which reduces its price (14). Thus, it may prove to be an efficient and cost-effective PD-1 inhibitor for the treatment of advanced CC, and this warrants further study. Our team is currently conducting a clinical trial of a combination of tislelizumab and anlotinib for the treatment of CC resistant to standard therapy (27). However, in general, we have little data on the use of tislelizumab for the treatment of CC.

In this study, we revealed that tislelizumab therapy for R/M CC patients showed promising antitumor activity and tolerable toxicity. In addition, we investigated potential biomarkers from regular blood tests for predicting the responses and clinical outcomes of R/M CC patients treated with tislelizumab. It was reported that pembrolizumab monotherapy attained an ORR of 12.2% and a DCR of 30.6% and achieved a median PFS of 2.1 months in advanced CC (12), and the efficacy was modest. Due to the disappointing responses to ICI monotherapy, several clinical trials are currently being conducted to assess the efficacy of ICI combination therapies. A study combing pembrolizumab plus GX-188E, a therapeutic DNA vaccine was reported an ORR of 42% and a DCR of 58% but a median PFS of 4.9 months (28). In the CLAP study, the ORR and median PFS were 55.6% and 8.8 months, respectively, in patients with advanced CC who received camrelizumab and apatinib (13). A phase II, single-arm prospective study showed an ORR and median PFS of 59.0% and 9.4 months, respectively, in patients with PD-L1-positive R/M CC receiving sintilimab and anlotinib (29). The recently approved ICI agents, cadonilimab treatment in R/M CC was showed that the ORR, median PFS, and median OS were 33%, 3.75 months, and 17.51 months, respectively (30). Our study revealed an ORR and median PFS of 39.1% (95% CI, 30.1–48.2) and 19.6 months, respectively, in R/M CC patients treated with tislelizumab monotherapy or combination therapy. Thus, we report a longer median PFS time than the clinical trials mentioned above.

We observed different response rates for certain subgroups that were stratified based on clinical characteristics. The ORR was significantly higher for patients with SCC than non-SCC patients, which was consistent with previously reported results (29). This was probably because higher expression levels of PD-L1 are observed in SCC patients than in patients with other histological types (9, 31), indicating that SCC is sensitive to PD-1 inhibitors. We also found that the use of dexamethasone significantly improved the antitumor response. However, some studies have shown that concurrent dexamethasone therapy is detrimental to immunotherapy, as it decreases the number of T lymphocytes by increasing apoptosis and weakening their functional capacities (32–34). Conversely, dexamethasone has also been shown to suppress T cell exhaustion and immune evasion by decreasing PD-L1 and indoleamine 2,3-dioxygenase 1 activity (35). In this study, dexamethasone was mainly used to alleviate chemotherapy-induced nausea and vomiting, which may have generated selection bias for the effects of dexamethasone on the response to PD-1 inhibitors. Therefore, further studies are required to confirm the effects of dexamethasone on PD-1 inhibitor therapy. We found that the ORR of patients treated with more than five cycles of tislelizumab was high, suggesting that tislelizumab therapy approaching six cycles may yield a good antitumor response.

The most common TRAE of tislelizumab therapy was anemia, which has been previously reported in patients treated with tislelizumab (25, 26, 36). Most events recorded were of grade 1 or 2 and no new safety concerns were identified. Only 10.4% of the enrolled patients needed to discontinue tislelizumab due to TRAEs. Therefore, tislelizumab combination therapy was generally well tolerated. Compared with the approved anti-angiogenesis reagents and ICIs in R/M CC, such as bevacizumab, pembrolizumab and cadonilimab, tislelizumab has the following advantages: 1) prolonged PFS time and durable antitumor response; 2) tolerated and manageable TRAEs, and patients with other underlying medical conditions can be treated with it; and 3) the price is cheaper and affordable for many women with CC. However, there is no efficacious method to select the patients who will benefit from tislelizumab.

Prognostic indicators of PD-1 inhibitors are urgently needed due to the high heterogeneity of efficacy and the heavy economic burden of the treatment. PD-L1 expression levels, TMB, and MSI status have been shown to be useful biomarkers for the selection of ICIs for several solid tumors (37). However, the predictive efficacy of PD-1 inhibitors in treating CC is disappointing. Measurement of the abovementioned biomarkers is also complex and expensive. Therefore, it is important to develop simple, widely applicable, and efficient biomarkers for determining which patients are likely to benefit from treatment with PD-1 inhibitors. Routine blood tests can indicate the state of inflammation and nutrition, which have been reported to affect the efficacy of immunotherapy (38) and have the advantages of being easily available and cost-effective. One study assessed the relationship between immunotherapy and hematological parameters, namely NLR, PLR, MLR, albumin-to-globulin ratio, alkaline phosphatase and lactate dehydrogenase (LDH) in CC patients, and found that high pretreatment NLR and LDH were independently correlated with poor survival (20). Thus, the predictive value of hematological parameters, especially those reflecting the inflammatory and nutritional status of patients with CC receiving PD-1 inhibitors, merits further study.

In this study, we assessed the clinical value of baseline hematological parameters in R/M CC patients treated with tislelizumab and revealed that the levels of pretreatment serum CRP and CAR were strongly correlated with the response to tislelizumab and the patients’ prognosis. High CRP and CAR levels were significantly associated with low ORRs. Multivariate analysis demonstrated that elevated CRP level was a significant independent predictor of a poor response to tislelizumab and a short PFS. In addition, the serum CRP level displayed the highest predictive value for the response (CR + PR) to tislelizumab. We also found that the baseline CAR was a significant and independent prognostic factor for PFS and OS, as patients with elevated CAR had a short PFS and OS after tislelizumab therapy. However, the pretreatment NLR showed no significant association with the response to PD-1 inhibitors or PFS, which was in contrast to the results of a previous study (20) and warrants further investigation. Overall, pretreatment serum CRP and CAR levels may be the promising biomarkers for predicting the response to treatment and prognosis of R/M CC patients treated with PD-1 inhibitors.

CRP, an acute phase protein, is released by hepatocytes during systemic inflammatory responses and is mainly induced by interleukin 6 (IL-6) (39). Serum CRP was reported to be a prognostic indicator in several solid tumors (40). Elevated CRP level was significantly associated with poor OS and PFS in patients with CC (41). Additionally, CRP plays an essential role in both the innate and adaptive immune systems (42). CRP was shown to inhibit the Th1 differentiation and promote the Th2 differentiation of naïve CD4+ T cells (43). Several studies demonstrated that high levels of baseline serum CRP were associated with poor responses to ICIs and clinical outcomes (44–46), which might result from the suppressed binding of CRP to T cells and the inhibition of the initial stage of T cell activation (47). Our study is the first to report the relationship between serum CRP levels and treatment of CC patients with PD-1 inhibitors, and our results are consistent with those of previous studies of other solid tumors.

Albumin is a nutritional marker and negative acute-phase protein that is downregulated under inflammatory conditions (48). CAR and the modified Glasgow and prognostic score, which comprises CRP and albumin concentrations, have been shown to have prognostic value for several cancers (49). Previously, we reported that the serum CAR was associated with poor survival in patients with stage IB–IIA human-papilloma-virus-positive CC (50). Additionally, CAR was shown to reflect the tumor microenvironment induced by the inflammatory response. Recent evidence indicated that elevated CAR was associated with poor prognosis after immunotherapy (22, 23), which is similar to the results of this study.

There are several limitations to our study. The most important is that it was limited by retrospective data. Some information may have been omitted or neglected, even though we attempted to identify all relevant clinical characteristics, treatments, and follow-up information. The efficacy and TRAEs of tislelizumab in R/M CC patients may, therefore, have been misestimated. Furthermore, CRP and CAR are not specific inflammatory indicators and may be affected by many factors. As they have the most value when measured during the stable stage of the disease, their utility in patients with R/M CC receiving tislelizumab or PD-1 inhibitors may have been overestimated.

In conclusion, our study demonstrated the tolerable toxicity and encouraging antitumor activity of tislelizumab in treating patients with R/M CC. Additionally, levels of pretreatment serum CRP and CAR were shown to predict the response to tislelizumab and the prognosis of patients with R/M CC. These results may help provide cost-effective PD-1 inhibitors to CC patients, prevent unnecessary therapy, and reduce the overall medical expenses. Additional investigations in the form of large, randomized controlled trials are warranted to fully understand this relationship.

## Data availability statement

The original contributions presented in the study are included in the article/**Supplementary Material**. Further inquiries can be directed to the corresponding authors.

## Ethics statement

The studies involving human participants were reviewed and approved by the Institutional Review Board of Sun Yat-sen University Cancer Center. Written informed consent for participation was not required for this study in accordance with the national legislation and the institutional requirements.

## Author contributions

MZ, XZ, HG and XC contributed to conception and design of the study. XZ, HG and XC completed the work of follow-up, performed the initial analyses and wrote the first draft of manuscript. BP, HX, MJ and SX collected the data. MZ critically reviewed and revised the manuscript. All authors contributed to the article and approved the submitted version.
