# Supplementary material for: Tislelizumab for cervical cancer: A retrospective study and analysis of correlative blood biomarkers
Source: Front Immunol. 2023 Feb 15;14:1113369. doi: 10.3389/fimmu.2023.1113369 (PMC9975598; doi:10.3389/fimmu.2023.1113369)

**Supplementary material**

**Supplementary figure legends**

**Figure S1.** Association between pretreatment GNRI and PLR and clinical outcomes in patients with R/M CC who underwent tislelizumab treatment. Kaplan–Meier curves of PFS and OS for GNRI (A, B) and PLR (C, D) in the enrolled population. Kaplan–Meier analysis and log-rank tests were used for comparison between low-level group (blue group) and high-level group (red group) of GNRI and PLR in PFS and OS. GNRI, geriatric nutritional risk index; PLR, platelet-to-lymphocyte ratio; PFS, progression-free survival; OS, overall survival; R/M CC, recurrent or metastatic cervical cancer.


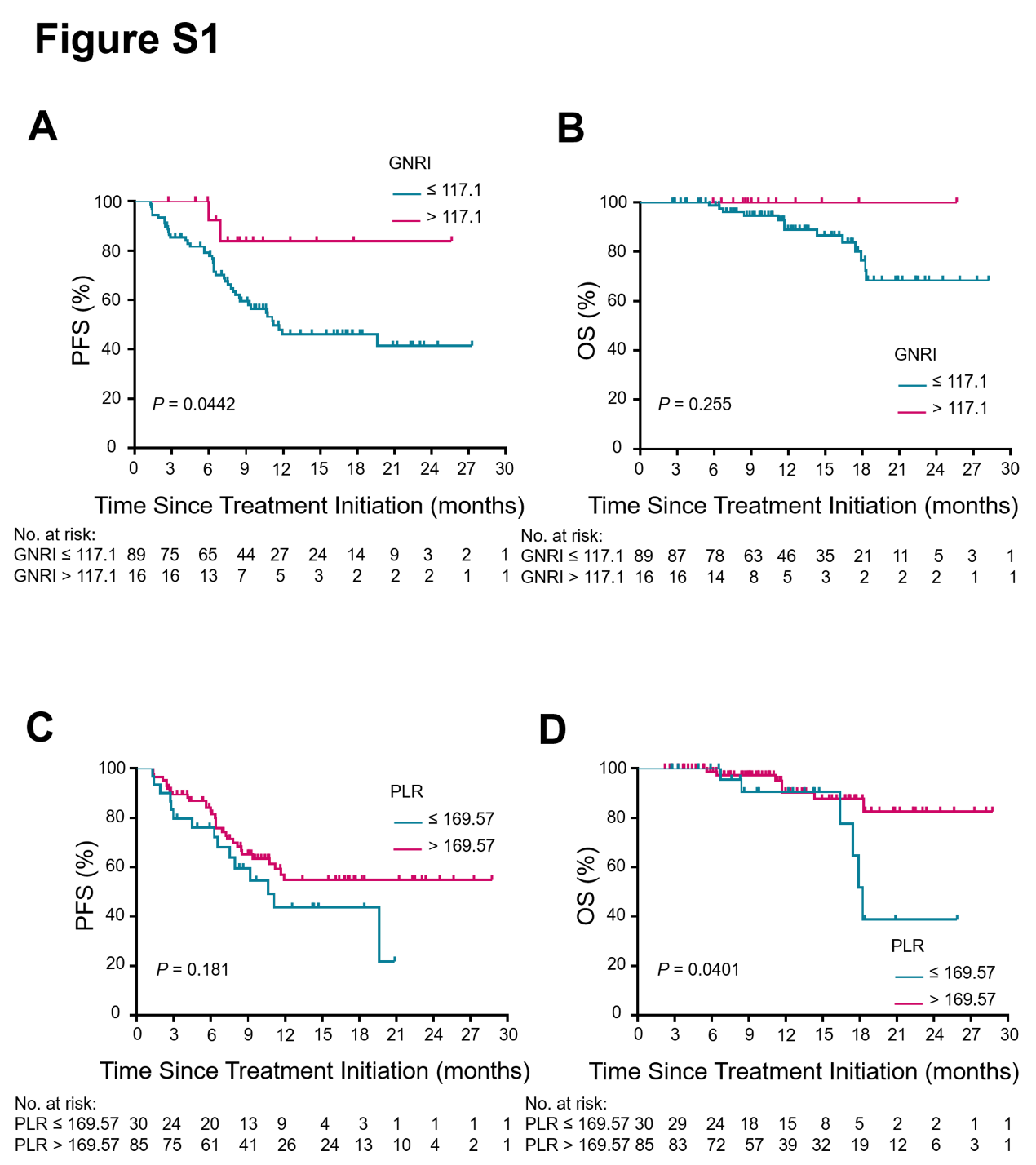

Supplement: Supplementary file 1 [file DataSheet_1.docx]
